# Dr. A. J. Volck's Seventieth Birthday

**Published:** 1898-06

**Authors:** 


### AMERICAN JOURNAL

—OF—

### DENTAL SCIENCE.

VOL. XXXII. BALTIMORE, JUNE, 1898. No. 2.

### Selections.

ARTICLE I.

### DR. A. J. VOLCK'S SEVENTIETH BIRTHDAY.

An interesting event in the history of the Association of Dental Surgeons of Baltimore was the complimentary dinner given to the President, Dr. Adalbert J. Volck, on Thursday, April 14, 1898, by his associates, in commemoration of his seventieth birthday. A permanent record of the occasion exists in a pamphlet issued shortly afterwards, containing the speeches and remarks delivered, from which the following is quoted.

An invitation was sent by those whose names are attached, and accepted by Dr. Volck:

BALTIMORE, March 16, 1898.

DR. ADALBERT J. VOLCK:

*Dear Doctor:*—The undersigned, members of the Association of Dental Surgeons, request the honor of your company at a complimentary banquet in commemoration of your seventieth birthday, April 14, 1898.

Your acceptance will be a mutual recognition of personal friendship, and will serve to continue and strengthen the evidences of good will and justice toward one another which have so notably distinguished the history of the Association in the ten years of its existence and which must be perpetuated if the objects—"the mental improvement of its members and the advancement of dental and oral surgery"—are to be attained.

Yours faithfully,

| | |
|---|---|
| WM. A. MILLS, | CHAS. E. DUCK, |
| E. E. CRUZEN, | JOS. G. HEUISLER, |
| M. GIST SYKES, | A. C. MCCURDY, |
| CLARENCE JONES GRIEVES, | H. A. WILSON, |
| CHAS. C. HARRIS, | S. LESLIE LECRON, |

RICHARD GRADY.

REMARKS OF DR. J. G. HEUISLER, VICE-PRESIDENT.

*Gentlemen :*

We meet this evening and tender this banquet to our respected President, Dr. Adalbert J. Volck, to celebrate the 70th anniversary of his birth. Papers and letters of congratulation will be read demonstrating to us the esteem and admiration in which he is held by his friends and contemporaries. To these testimonials it would be superfluous for me to attempt to add anything, In the name of our little society, I welcome him most heartily, and feel that I voice the sentiments of all. We congratulate him upon his birthday and wish him many more years of happiness ane usefulness.

"Sir, you are very welcome . . .
It must appear in other ways than words,
Therefore I scant this breathing courtesy."

The following is a list of speakers and topics after dinner :

TOASTS.

1. To our Guest, the President of the Association.

"Not chance of birth or place has made us friends,
But the endeavor for the self-same ends,
With the same hopes, and fears, and aspirations."

—Dr. Volck.

2. The life and work of Dr. Volck.

"We speak his praise who wears to-day
The glory of his seventy years."

—Dr. Grady.

3. Reminiscences of Dr. Volck's Boyhood.

"I watch them drift the old familiar faces."

—Dr. Grieves.

4. Dr. Volck as a dentist.

"Strong in will
To strive, to seek, to find, and not to yield."

—Dr. Harris.

5. Dr. Volck as a man.

"Speak of me as I am." —Dr. Wilson.

6. Dr. Volck as an artist.

"All passes; Art alone
Enduring stays to us;
The bust outlasts the throne,
The coin, Tiberius."

—Dr. Sykes.

7. Poem. —Dr. Mills.

When the fitting time arrived, the toastmaster rose and spoke as follows :

### REMARKS OF DR. C. E. DUCK.

GENTLEMEN :—We are gathered here to-night with overflowing hearts to give honor to our beloved friend and President, Dr. Volck, who has happily lived to realize the truth of Dr. Oliver Wendell Holmes' words, that it is better to be young seventy than old fifty. It has been my happy privilege to know Dr. Volck for nearly thirty-five years, and in all this time I have always found him the kind friend, the good adviser, the able practitioner, free from all petty jealousy and ever willing and ready to take a firm stand for the right.

I was once a student of Dr. Volck, and had I then the wisdom, which unfortunately only comes with experience, I might have obtained knowledge which if I have ever gained has been after much labor and many bitter failures. Those who *really* know our worthy President, have, I am sure, always found him ready to impart to others the knowledge which he has gained only after years of labor and experience. He has always been the sympathizing friend, the earnest worker, envious of none, with a word of encouragement for every conscientious co-laborer.

We thank God that to night we find him strong both in mind and body with apparently many more years of usefulness before him; and we trust and believe that his future reward will be commensurate "with his many good deeds done in the body."

RESPONSE.—Dr. A. J. Volck.

GENTLEMEN:—If I say my heart is touched to its utmost depth, by this evidence of your friendship and respect, it conveys only a meagre expression of the feelings permeating me on this occasion.

It was such a surprise to me, when I was told that I was to be the recipient of such an honor, as this banquet represents, that I could not, and cannot now, understand, by what merit I should be entitled to it. I can only see in it, the effort of that pleasant brotherly tone that prevails amongst the members of this association, since its beginning, and of sympathy for a man who has borne the burden of professional labor for so many years, without (may I be permitted to say) ever having disgraced his profession. I have seen our profession in this town represented by a small number of men—less than the fingers of your hands—I have seen it grow to a vast body of hard working, hard thinking men, an honor and a blessing to the community in which they live. I can recall one by one the marked men, like sparks of light amongst the great mass.

I am conscious of the thousands of students, who have received their instruction among us, and I feel right proud to stand here the oldest worker of them all, thank God, in the vigor of undimished usefulness, capable of appreciation and joining in the foremost march of this honest, useful army.

I have seen their work and I have seen also their dissentions. Men, now and then, have disturbed the peace of the profession by their selfish ambitions, but this is a drawback any large profession has to contend with. But I have never seen anything of this kind amongst us here. There is not blot or blemish on the brotherly unison of the members of this association. I have never met them but to feel at home amongst them, never met them but my stock of knowledge was increased, never left them without a renewed feeling of respect for them, without feeling they respected me.

From my heart, gentlemen, I thank you for this honor. It is a good thing to feel at the close of your career that you have your fellows' sympathy, that they forget your past weaknesses, and cover your sins with the mantle of sweet charity.

The following toast was then announced, and Dr. Richard Grady was called upon to respond :

THE LIFE AND WORK OF DR. A. J. VOLCK.

"We are here to-night," said Dr. Grady, "to show the feeling of the Association of Dental Surgeons toward our distinguished guest, the Nestor of the dental profession in Baltimore. We join in making memorable the 70th anniversary of the birth of our president, Doctor Adalbert J. Volck, whose practice extends farther back into the century than any practitioner in Baltimore. Born in a foreign land, the career at first planned for him was far other than the scenes in which it has been cast. His father, Andreas Volck, a large manufacturing chemist of Nuremberg, Bavaria, was a man of wide culture and learning who devoted his leisure hours to scientific researches, especially in the field of theological learning. Several of his sons are men of renown in various fields of human activity in their native land, only two of them having come to America.

Adalbert J. Volck was born in the City of Augsberg, Bavaria, April 14, 1828. While yet in early childhood, his parents removed to Nuremberg, where his early education was secured, after which he became a student in the University of Munich. His career here was cut short by reason of having to flee the country for being suspected of sympathizing with the revolution of 1848 when Shurz, Hecker and many others of our now distinguished American citizens inaugurated a movement for greater freedom, that resulted in their expatriation. Escaping from the Fatherland, our fleeing student made his way in secret to Bremen, whence he sailed for New York, where he landed late in November, 1848.

A penniless stranger in a strange land, little familiar with a foreign tongue, he met with discouragement every where he applied for work. A young foreigner found little demand for an expert chemist or one of scientific attainments. He sought positions as teacher, as assistant, as chemist—anything that would bring him remuneration, but nothing was to be found. Hoping for better fortune in Boston, he departed for that city, where it was worse than in New York. He nearly starved. To sustain life he sought any kind of employment, manual or otherwise, but met with discouragement on every hand. By good fortune he came to the notice of Dr. Keep, an eminent dentist of Boston, and was given employment assisting him in chemical experiments and operations in his laboratory, and was here employed upward of a year.

Dr. Chapin A. Harris hearing of the young German, sent for him to assist in his office and the laboratory of the Baltimore College of Dental Surgery. He became at once a teacher as well as student in the dental school, and on graduating in 1851, opened an office in Baltimore, where he has since been in active practice for upwards of 45 years. Though unable to remain at the University in Germany to complete his course, Dr. Volck wrote his thesis here, and had conferred upon him the degree of Ph. D. He has always been an original investigator, never being content to tread the beaten paths of others' research. He made many investigations in the field of microscopy with Dr. Christopher Johnson. In metallurgy he has had a wide experience, assaying thousands of samples of foil used in the practice of dentistry to establish what mauufacturers' products are pure and in which an alloy is found, either by the carelessness or design of the manufacturer.

Dr. Volck was the first dentist to make use of enamel inlays for fillings, as far back as 1854. At that time the work was inlaid in gold, and was more durable than more modern methods of cement. The doctor now has in his possession his first specimen of work and the inlay is in a perfect state of preservation.

The doctor works at his profession for the nourishment of his body, finding food for his soul in art. And it is not in one branch of art alone that this many sided man excels. Had he chosen art for his life-work instead of dentistry, there can be little doubt he would have made his mark. As a designer and draughtsman he is excelled by few; as a painter he shows a familiarity with the handling of pigments and of the various schools of art and the individual styles of the old masters that might be envied by many who make a profession of painting. The branch of art, for which the doctor is most widely known, perhaps, is his fine work in metals. There is probably not his superior in the establishments of Tiffany or Graham in New York, or in any of the cities of America. The Apold Testimonial and the Hooper Testimonial, the products of his brain and hand, are as artistic pieces of work as one can find. For exquisite and artistic delicacy of design, and for boldness of execution, they could not well be excelled. Much of the finest work turned out by the various establishments of artistic metal work in Baltimore, is from his designs.

When the work of the day is over—when his last patient has gone—then the work of his heart and soul begins. His hours are long and midnight usually finds him in his laboratory with the tools of the silversmith in his hand, or the charcoal or pencil af the artist. Though at the seventieth milestone in the journey of life, Dr. Volck retains to a remarkable degree his faculties. In his professional and private life he is revered by his colleagues and loved by all his friends.

> "Thanks, thanks to thee, my worthy friend,
> For the lessons thou hast taught."

Dr. C. C. Harris then responded to

DR. VOLCK AS A DENTIST,

Dr. Volck stands before us as the one representative of dentistry in this State from the time of its first recognition as a profession, when the world's first dental school had just become fairly well established under a corps of intelligent instructors. It was at this time he was induced to come to Baltimore by Dr. Chapin A. Harris to teach in the College and make porcelain teeth, for which purpose he was the foremost and best suited in all the country. Having given special attention to chemistry in Harvard University, he soon found himself devoting much time to the coloring and production of artificial or porcelain teeth, and was so employed for some time before attracting the attention of Dr. Harris.

The blocks made in those days were very different from the ones in use to-day, and were riveted to metal plates. Dr. Volck received gold medals for proficiency in making blocks.

While acting in the capacity of semi-professor, he took the regular course in order to graduate. The professors decided him "satisfactory" without requiring the usual examinations. Amalgam was known in those days but considered so common that Prof. Cone demanded of all students a sacred obligation or promise never to use it.

Dr. Volck was associated in practice with such well-known men as Harris, Cone and Blandy. Through Dr. Harris he met and knew intimately most of the distinguished practitioners of that time.

In 1851 ho opened an office for himself, from which time to the present he has enjoyed a rich and distinguished practice. Baltimore only had a half dozen good dentists in those days, among which were Harris, Mickle, Cone and Cherry. Dr. Volck was the first person to put in a porcelain filling, which was done by grinding the porcelain to fit the cavity and fasten it in position by filling around it with gold. This was done in 1854.

Dr. Volck enjoyed an intimate acquaintance with Dr. Maynard and states the latter to be without a superior, giving him the credit of being the first to fill roots with gold.

Dr. Volck has devoted much time to microscopy, and worked in this line for years with Dr. Christopher Johnson. Their work on dental anatomy produced many important observations, which were given to the profession. The Doctor has always been foremost in taking hold and putting into practice of new and worthy developments, and to-day is actively engaged in modern crown and bridge-work. Always being a scientific man he puts into practice all the late ideas of antiseptics and thinks nothing too new to be worthy of consideration. It is thus we find him among the most advanced dentists of the present day.

It has always been a happy characteristic of Dr. Volck to lend assistance and give the benefit of his vast experience to brother practitioners. In this way he has drawn around him a coterie of warm professional friends, who will hold him in reverence and admiration.

May this seventieth birthday be followed with renewed vigor and energies, that we may all meet to do him honor at the end of four score years.

Dr. C. J. Grieves then read the following!

EARLY REMINISCENCES OF DR. A. J. VOLCK.

BEL AIR, MD., March 8th, 1898.

DR. RICHARD GRADY, Baltimore, Md.,

My Dear Sir :—Very gladly would I comply to write at length "reminiscences from childhood's early hours," respecting my good friend, Dr. Volck, if there was not a lamentable scarcity of material, so far as my own acquaintance with him in that far off period is concerned. I sincerely regret the fact, the more so because a contribution of this character would in all probability help in filling up an interesting part in a life, the greater portion of which has been spent in this country, and conspicuously identified with its interests, history and welfare.

It was over fifty-three years ago that some three miles east of the renowned city of Nuremberg, towards the Franconian Mountains, and in a region of unobtrusive picturesqueness, on the River Pegnitz, a colony of artists had made their home, under the leadership of Mr. Enzing-Muller, a man of original mind, a vigorous thinker, a skilful engraver deservedly acknowledged, also a composer of elaborate epic subjects, and striking allegories. With him came from Munich a younger man, a former pupil of the renowned Wilhelm von Roulbach, Johann Buchner, who as an artist had the gift of good coloring, that Mr. Enzing-Muller did not share. Together with his brother Charles, and four young men, the students of Dr. Enzing-Muller, and all practising steel-engraving, these were the members of that colony which Dr. Volck, then—as I believe—studying in the Nuremberg Polytechnic School, came out to visit from time to time. From this period dates my acquaintance with him. Recollection, in the absence of written data, gives me only general facts of his visits, with one exception, namely that he taught us younger men how to make a thermometer, whereat we fell to work industriously to manufacture for ourselves and friends these useful recorders of temperature. Young Volck was nearly five years the junior of myself and room-mate, Fridolin Schlegel, who many years ago has returned to Germany.

Very soon after that period, during which occurred the visits of young Volck, we all were engrossed with plans for emigrating to America, and in consequence I lost sight of him, ignorant of his being even alive, until we met again in Baltimore last winter, strangers in looks to one another, until names were exchanged and so the old ties joyfully renewedi I had indeed heard of him frequently—of his wonderful work in metal, his self-taught skill and inventive thought that recalled the great masters of our native city, Vischer and Adam Kraft, and I determined on my first visit to Baltimore to go and meet the man myself, and most pleasant has been the meeting. Of all that little colony of artists and students there is but one more beside myself alive, and I count it a singular good fortune, after more than half a century, to renew a friendship calling up so many dearly cherished ties of home, of kindred and associations, in the person of one honored alike for his genius as an artist and his sterling qualities as a man.

Yours very sincerely,

JOHANNES A. OERTEL.

Dr. H. A. Wilson read :

### DR. VOLCK AS A MAN.

Away back in the Forties, there came amongst us a young artist of excellent, old family, seeking fame, if not fortune, on our shores. With varying success, through years of industry and patient toil, he won his way to the hearts and homes of our people, who recognized his exalted genius and his worth.

During the Civil War his sympathies were deeply enlisted in behalf of the struggling South; and few of us suffered greater pecuniary losses or made more sacrifices of time and labor, whilst his gifted brush and pencil were employed in illustrating scenes and incidents of that exciting period. His exquisite poetic taste in design and execution of his works is acknowledged by all. Of rude exterior, with a countenance and deportment expressive of his sturdy, manly character, he has lived among us these many years in sunshine and adversity, enjoying the respect and admiration of the whole community.

Ever ready to help the afflicted and the poor, his responsive heart was always touched by the cry of distress and his slender means were freely offered with characteristic modesty. With a disposition as guileless and tender as a child, with a sentiment of loyal devotion to every duty imposed upon him, his many friends are proud to claim him as a brother. And now that he is fast nearing the call "to cross over to the other side," it is fitting that on this occasion of his passing the 70th milestone we should give utterance to our feeling for Dr. Adelbert J. Volck.

BALTIMORE, March 26th, 1898. JAMAS HOWARD.

### DR. VOLCK AS AN ARTIST.

(Read by Dr. M. Gist Sykes.)

Dr. Volck is an artist in every sense of the word. I have had the pleasure of his acquaintance for the past forty years; during this long period I have seen much of his art work, pencil and chisel, and consider him a man of great talents in every line of art. His later work in silver has shown our people his capability in this line of art. May he live long to enjoy the fame he has so justly won in this community.

Yours truly,

THOS. HEDIAN.

### TO DR. A. J. VOLCK.

(On his seventieth birthday.)

On this thy natal day, we would conspire
To grasp thy hand, with tenderness, old friend;
And wish for thee thy every heart's desire,
With every boon a gracious God may send
Of friendship, love and everything that cheers,
To help thee bear thy weight of Seventy years.

We greet thee with affection most sincere,
As one who e'er has been our honored guide;
Whose teachings in the past, we all revere,
And for all future time will point with pride
To each event, which thee, to us, endears,
and marks an epoch in thy Seventy years!

Again, we greet thee as our honored guest,
And glad, indeed, are we to be thine host;
And we would make of thee but one request,
Which is to join us in our heartsome toast:
"Through joys or sorrows, aye, through smiles or tears,
God give thee strength unto thy Fourscore years!"

W. M. P.

Remarks of Dr. Wm. A. Mills:

GENTLEMEN: Having the honor to be one of the charter members of the Association of Dental Surgeons, I have watched with the greatest pleasure its course of events, from month to month, and from year to year, and so far they have been an unbroken flow of success and progress.

Never has the occasion arisen, but that I feel greatly honored in having my name enrolled with its fellows. From the earliest history of the organization, its membership has been composed of some of the brainest, and most highly respected and progressive dental practitioners in the city.

In respect to its social feature, we have faithfully followed the scriptural admonition, "let brethren dwell together in unity," and "love ye one another."

For this, we owe much gratitude to its founder, Dr. Richard Grady; also for a never-ceasing fountain of knowledge of things in general, and dentistry in particular, to our revered associate, Dr. A. J. Volck, our guest for the evening.

### AN ACROSTIC.

Dear Friend, on this thy natal day,
*Righteousness* and *peace* be thine we pray,
And may they abide till thou art hoary.

*Joy* fill thy pathway, and may at last
*Victory* be written of the past
On every line of thy life's story.

*Lord*, bless, we pray, our beloved friend,
Care for him, until his life shall end;
Keep him steadfast; Thine be all the *Glory*.
Amen. W. A. M.

### REMARKS OF DR. A. C. MCCURDY.

*Mr. President and Gentlemen*:

It was through friendship, esteem and respect I have for our honored president and friend, Dr. Volck, that I come here from the country on this disagreeable and stormy night to pay my respects to him. I always enjoyed attending the meetings especially when our old friend was present. It was instructive and entertaining to hear him give his experience before and after he came to America, especially his career as a dental practitioner in the days of its infancy. I have never been in his company but after I left him have thought what I had learned. His brain always seemed to be a store house of knowledge. When I left home to come here I did not expect to be called on to respond to a toast nor did I expect to make an address; therefore I am not prepared. I simply came to pay my respects to our honored friend, Dr. Volck on this his 70th birthday, and I hope he will spend many more pleasant nights with us around the festive board.

